# Upregulation of minichromosome maintenance complex component 3 during epithelial-to-mesenchymal transition in human prostate cancer

**DOI:** 10.18632/oncotarget.16835

**Published:** 2017-04-05

**Authors:** Paul A. Stewart, Zahraa I. Khamis, Haiyen E. Zhau, Peng Duan, Quanlin Li, Leland W.K. Chung, Qing-Xiang Amy Sang

**Affiliations:** ^1^ Department of Chemistry & Biochemistry, Florida State University, Tallahassee, FL, United States of America; ^2^ Samuel Oschin Comprehensive Cancer Institute, Cedars-Sinai Medical Center, Los Angeles, CA, United States of America; ^3^ Institute of Molecular Biophysics, Florida State University, Tallahassee, FL, United States of America; ^4^ Laboratory of Cancer Biology and Molecular Immunology, Department of Biochemistry, Faculty of Sciences, Lebanese University, Beirut, Lebanon

**Keywords:** epithelial-to-mesenchymal transition, minichromosome maintenance complex proteins, metastasis, androgen receptor, proteomics

## Abstract

Metastasis is often associated with epithelial-to-mesenchymal transition (EMT). To understand the molecular mechanisms of this process, we conducted proteomic analysis of androgen-repressed cancer of the prostate (ARCaP), an experimental model of metastatic human prostate cancer. The protein signatures of epithelial (ARCaP_E_) and mesenchymal (ARCaP_M_) cells were consistent with their phenotypes. Importantly, the expression of mini-chromosome maintenance 3 (MCM3) protein, a crucial subunit of DNA helicase, was significantly higher in ARCaP_M_ cells than that of ARCaP_E_ cells. This increased MCM3 protein expression level was verified using Western blot analysis of the ARCaP cell lineages. Furthermore, immunohistochemical analysis of MCM3 protein levels in human prostate tissue specimens showed elevated expression in bone metastasis and advanced human prostate cancer tissue samples. Subcutaneous injection experiments using ARCaP_E_ and ARCaP_M_ cells in a mouse model also revealed increased MCM3 protein levels in mesenchymal-derived tumors. This study identifies MCM3 as an upregulated molecule in mesenchymal phenotype of human prostate cancer cells and advanced human prostate cancer specimens, suggesting MCM3 may be a new potential drug target for prostate cancer treatment.

## INTRODUCTION

Prostate cancer (PCa) is the most common non-skin malignancy and a major leading cause of cancer deaths among men in the Western world. An estimated 180,000 men would be diagnosed with prostate cancer in 2016 with 26,000 estimated deaths occurring from the disease in the USA [1]. Most patients who die from prostate cancer develop lethal castration-resistant or androgen independent metastatic disease that fails to respond to available therapeutics. A mechanistic understanding of the metastatic cascade can help identify new treatment strategies, identify patients with aggressive lesions, and control the dissemination of cancer cells to other parts of the body to improve prostate cancer patient survival. Metastasis of tumor cells to distant sites is believed to involve epithelial-to-mesenchymal transition (EMT) by which immotile epithelial cells become motile, aggressive, and metastatic [2]. In prostate cancer, EMT has been described as a notable feature of the androgen repressed prostate cancer (ARCaP_E_/ARCaP_M_) cell line model. This ARCaP prostate cancer cell line not only represents a relevant EMT model but also mimics the clinical course of prostate cancer metastasis. The epithelial-like (ARCaP_E_) cells are isolated from parental ARCaP cells and induced to transition to mesenchymal phenotype (ARCaP_M_) upon exposure to soluble growth factors or surrounding skeletal microenvironment [3]. Recent studies highlight the molecular and behavioral signatures of this ARCaP EMT model [4, 5]. Understanding the molecular basis of EMT will provide indispensable insights into the metastatic process and valuable prognostic and therapeutic targets.

Minichromosome maintenance proteins encompass a group of ten factors that function in initiation and elongation of DNA replication [6]. MCM1 and MCM10 do not belong to this family but also play a role in DNA synthesis. MCM1 is a transcription factor that can affect DNA synthesis [7], and MCM10 functions in initiation of DNA replication [8]. While MCM8 is presumed to possess a helicase activity during elongation of DNA replication [9], the role of MCM9 has not yet been addressed [10]. MCM2-7 are six structurally related proteins crucial for initiation of DNA replication. During late M/early G1 phases, inactive MCM2-7 proteins are recruited to DNA and loaded on replication origins as a double hexamer to preserve bi-directionality of replication forks [11, 12]. At this point, the six MCM subunits associate with other proteins to form the pre-replication complex. In S phase, the double hexamer interacts with the pre-initiation complex proteins and serves as a part of the replicative helicase unwinding the DNA and “licensing” the origin to undergo precisely one replication per one cell cycle round [13]. Recently, Frigola *et al*. showed that MCM3 is indispensable and sufficient to affect recruitment and loading of the double hexamer on DNA [12]. This MCM subunit is used as a proliferation marker that can determine tumor growth propensities. Elevated expression of MCM3 is also correlated with poor prognosis and more advanced stages in ovarian cancer, squamous cell carcinoma, and malignant melanoma [14–16]. These findings underscore the function of MCM3 in cancer progression and suggest a putative role of this protein in prostate cancer metastasis.

This study aims to further understand the molecular mechanisms of prostate cancer metastasis using an ARCaP cell line EMT model, to find new targets for the development of more effective treatment strategies, and to improve clinical outcomes of prostate cancer patients. Biochemical analyses of our EMT model revealed increased expression of MCM3 upon EMT in the ARCaP cell line model and in human metastatic prostate tissues. This finding suggests a pivotal role of MCM3 during EMT and prostate cancer metastasis.

## RESULTS

### Protein expression profile in the ARCaP EMT model

Proteomic analysis was conducted to identify EMT related changes in protein expression in the ARCaP human prostate cancer cell line model. A set of 1,152 proteins were identified in ARCaP_E_ and ARCaP_M_ in our proteomics approach (Supplementary Table 1). Only 64 proteins were differentially expressed between the two cell lines with a P-value < 0.05 and a fold-change of at least 1.5 (Supplementary Table 1). A total of 35 proteins were upregulated and 29 proteins were downregulated in ARCaP_M_ compared to ARCaP_E_. A selection of the proteins with the highest fold-changes is listed in Table 1. Consistent with their respective phenotypes, ARCaP_E_ expressed significantly higher levels of epithelial markers such as tight junction protein TJP1 (P = 0.004, -2.69 log_2_ fold-change; also known as ZO-1) and keratins KRT1 and KRT8 (P = 0.005, -1.95 log_2_ fold-change; P = 0.024, -1.32 log_2_ fold-change, respectively). The ARCaP_M_ upregulated proteins were predominantly correlated with the mesenchymal and aggressive phenotype. They expressed higher levels of vimentin (VIM), matrix metalloproteinase 1 (MMP1) and major vault protein (MVP). The expression of the mesenchymal marker vimentin was not significant (P = 0.09, 0.72 log2 fold-change; Supplementary Table 1), but was in agreement with a previous study of ARCaP_E_ and ARCaP_M_ (0.85 log_2_ fold-change) [17]. The elevated expression of matrix metalloproteinase 1 in ARCaP_M_ (MMP1; P = 0.036, 1.61 log_2_ fold-change) is consistent with the functions attributed to this metalloprotease in prostate cancer progression and metastasis [18]. ARCaP_M_ showed high levels of major vault protein (MVP or LRP) (P = 0.002, 1.41 log2 fold-change), a protein that is increased in advanced prostate cancer and expressed in drug resistant cell lines and various types of cancers [19, 20].

**Table 1:** Differentially expressed proteins between ARCaP_E_ and ARCaP_M_

Gene Symbol	Log_2_ Fold-change	T-test P-Value
RBM3	1.72	0.002
MMP1	1.61	0.036
MCM3	1.49	0.015
MVP	1.41	0.002
MCM7	1.40	0.048
MCM6	1.07	0.047
KRT8	−1.32	0.024
KRT1	−1.95	0.005
TJP1	−2.69	0.004

Log_2_ fold-change is calculated as log_2_ (ARCaP_M_/ARCaP_E_).

Abbreviations: RBM3, RNA binding motif protein 3; MMP1, matrix metalloproteinase-1; MCM3, minichromosome maintenance complex component 3; MVP, major vault protein; MCM7, minichromosome maintenance complex component 7; MCM6, minichromosome maintenance complex component 6; KRT8, keratin 8; KRT1, keratin 1; TJP1, tight junction protein 1.

### Pathway analysis of differentially expressed proteins

To gain mechanistic insight into the underlying biology of ARCaP_E_ and ARCaP_M_ cells, altered proteins (at least 1.5 fold higher in either cell line) were input into GeneGO MetaCore (Thomson Reuters, https://portal.genego.com/) for pathway analysis, and a number of pathways were enriched (false discovery rate or FDR < 0.05) (Table 2, Supplementary Table 2). Four of the top 10 pathways were related to cell adhesion and two were related to cell cycle. One of the significantly altered pathways was *Cell cycle_start of DNA replication in early S phase* (pathway FDR = 0.0060; Table 2), which included MCM3, MCM6, and MCM7. All of these MCM family members were differentially and highly expressed in ARCaP_M_ compared to ARCaP_E_ (Table 1).

**Table 2:** Top altered pathways between ARCaP_E_ and ARCaP_M_

Pathway	Proteins Found in Pathway	FDR*
Cell adhesion_Role of tetraspanins in the integrin-mediated cell adhesion	8	0.0004
Cell cycle_Chromosome condensation in prometaphase	6	0.0009
Cytoskeleton remodeling_Keratin filaments	7	0.0014
Cell cycle_Start of DNA replication in early S phase	6	0.0060
Blood coagulation_Platelet microparticle generation	8	0.0104
Cell adhesion_Endothelial cell contacts by non-junctional mechanisms	5	0.0104
Cell adhesion_Chemokines and adhesion	9	0.0181
Neurophysiological process_Receptor-mediated axon growth repulsion	6	0.0214
wtCFTR and deltaF508 traffic/Membrane expression (normal and CF)	6	0.0215
Cell adhesion_Alpha-4 integrins in cell migration and adhesion	5	0.0312

*FDR: False discovery rate or adjusted p-value.

### Overexpression of MCM3 in ARCaP_M_ cells and human prostate cancer tissues

**Figure 1:**
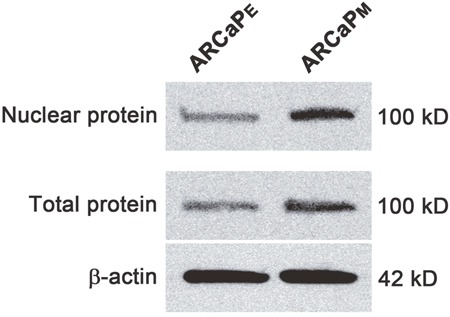
Western blot of MCM3 in ARCaP_E_ and ARCaP_M_ cells Total and nuclear proteins were analyzed for MCM3 expression. Beta-actin was used as a control.

**Figure 2:**
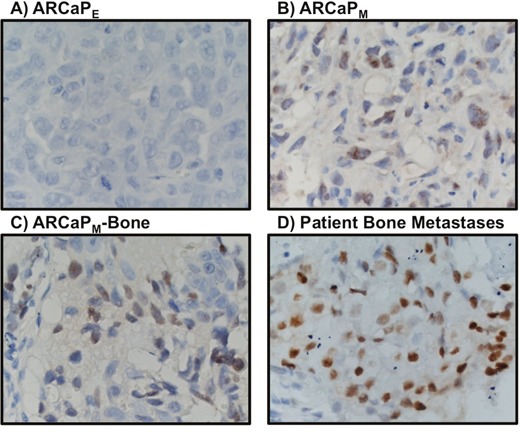
MCM3 expression profiling in ARCaP_E_ cells **(A)**, ARCaP_M_ cells **(B)**, ARCaP_M-Bone_ cells **(C)** and patient bone metastasis **(D)**. MCM3 expression is higher in primary tumor induced by subcutaneous injection of ARCaP_M_ than by ARCaP_E_ **(A-B)**. MCM3 is highly expressed in ARCaP_M-bone_ **(C)** and in three out of four human bone metastatic tissues from prostate cancer patients **(D)**. Images were taken at 400X magnification.

Interestingly, upregulation of three of the six homologous MCM subunits, MCM3 (P = 0.015, 1.49 log2 fold-change), MCM6 (P = 0.047, 1.07 log2 fold-change), and MCM7 (P = 0.048, 1.40 log2 fold-change) proteins was observed in ARCaP_M_ cells. MCM3 showed the most significant difference and was further pursued. Western blotting analyses of total and nuclear proteins confirmed the abundant expression of MCM3 in ARCaP_M_ cells (Figure 1). These results were validated *in vivo* using immunohistochemical analysis of tumors generated from subcutaneous injection of ARCaP_E_, ARCaP_M_, and ARCaP_M-Bone_ into nude mice (Figure 2). ARCaP _M-Bone_ and ARCaP_M-C2_ cells are derived from ARCaP_M_ cell-induced bone metastatic tissues after two rounds of intracardiac inoculation of athymic mice [21]. Compared to ARCaP_E_ tumors, ARCaP_M_ and ARCaP_M-Bone_ tumors displayed higher levels of MCM3 protein. These data suggest that MCM3 upregulation is correlated with the increased *in vivo* metastatic ability of the ARCaP EMT model.

**Figure 3:**
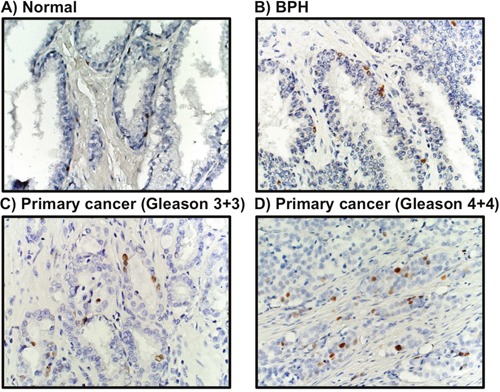
MCM3 expression in human prostate cancer MCM3 expression was visually absent in 12 normal prostate tissues **(A)**, barely expressed in 6 benign prostatic hyperplasia tissues **(B)**, and significantly increased in 12 prostate cancer clinical specimens **(C-D)**. Images were taken at 200X magnification.

**Figure 4:**
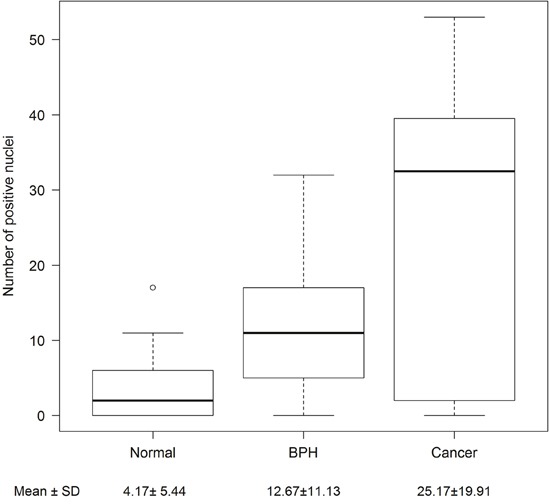
Semi-quantitative analysis of MCM3 expression in human samples The number of MCM3 positive nuclei from normal (n = 12), benign (n = 6), and cancerous (n = 12) patient tissue samples were counted in ten random 20X microscopic fields. Differences between normal, benign, and cancerous samples were determined using a Poisson regression model. Normal prostate and benign prostatic hyperplasia showed no significant difference in expression (p = 0.0866). MCM3 differential expression is significant between benign and cancer (p=0.01) and normal and cancer (p = 0.0001). Mean and standard deviations are shown.

Additional *in vivo* validation of MCM3 results was sought in a small cohort of metastatic human bone tissue specimens. Consistently, three out of four human metastatic prostate tissues showed significant high expression of MCM3 (Figure 2D). To assess the significance of our ARCaP EMT model for clinical prostate cancer progression, we evaluated the expression of MCM3 in normal prostate (n = 12), benign prostatic hyperplasia (BPH, n = 6), and primary prostate carcinoma tissues (n = 12). MCM3 was observed to be absent in normal prostate, barely observed in benign tumor, and highly expressed in late stage prostate cancer (Figure 3). Semi-quantitative analysis of MCM3 expression in the different human prostate tissues showed the lowest MCM3 expression in normal tissue and the highest in high-grade prostate cancer. There was a significant difference in expression between normal and cancerous prostate (P = 0.0001), BPH and cancer (P = 0.01), but not between normal and BPH (P = 0.0866) (Figure 4). These data further supported our findings of a positive correlation between elevated MCM3 protein levels and increased aggressiveness of the disease.

## DISCUSSION

Despite recent advances in prognosis, diagnosis and treatment of prostate cancer, development of lethal skeletal metastases remains a clinical challenge. Current treatment strategies are directed at patients with localized prostate carcinomas and offer only modest remission for those with more aggressive lesions [22]. We urgently need to understand the mechanisms by which prostate tumor cells disseminate to distant sites. Given the association of metastasis with EMT, it stands to reason that dissecting the molecular signatures of the EMT process will reveal valuable targets for prostate cancer therapy and clinical research. We used the ARCaP human prostate cancer cell lineage as an experimental EMT model of clinical prostate cancer metastasis [3]. ARCaP_E_ and ARCaP_M_ cells are derived from genetically identical cells isolated from the same human prostate cancer patient with bone metastases [3, 23, 24]. However, they exhibit different morphology, gene expression profiles and behavioral characteristics. ARCaP_M_ cells have increased expression of mesenchymal markers such as vimentin, N-cadherin, and RANKL; decreased epithelial markers like E-cadherin; increased proliferation compared to ARCaP_E_; and higher propensity for bone metastasis [3, 23, 25]. Conversely, ARCaP_E_ cells display epithelial morphology with lower invasion, migration, growth rate, and metastatic potential [3, 23, 25]. Both ARCaP_E_ and ARCaP_M_ cells are derived from the parental ARCaP cells which express low levels of androgen receptor and prostate specific antigen, and are repressed by androgens [24].

Our study showed elevated expression of three members of the MCM family of proteins, MCM3, MCM6, and MCM7, in ARCaP_M_ as compared to ARCaP_E_ cells. While MCM6 was reported to be prognostic marker in a few malignancies [26], MCM7 is extensively studied in human prostate cancer tissues and is correlated with higher propensities for prostate cancer progression, invasion, and metastasis [27, 28]. We selected MCM3 for further study based on lack of reports in the literature on the expression of MCM3 in prostate cancer, and on its critical importance in the cell cycle. In our proteomics and Western blotting analyses, MCM3 was upregulated upon EMT in the ARCaP prostate cancer cell model (Figure 1). This upregulation corroborated the *in vivo* investigation of tumors generated from subcutaneous injection of ARCaP_E_, ARCaP_M_, and ARCaP_M-Bone_ (Figure 2) in nude mice. To investigate the clinical significance of MCM3 in human prostate cancer progression, immunohistochemical analysis was performed on normal human prostate, benign prostatic hyperplasia, primary prostate cancer, and human bone metastatic prostate cancer tissues (Figures 2D and 3). In accord with our previous results, expression of MCM3 was significantly increased in primary tumors and further upregulated in bone metastases, suggesting a pivotal role of MCM3 in prostate cancer progression and metastasis.

In the majority of cases observed in the clinic, human prostate cancer progression proceeds from an androgen-dependent to an androgen-independent state. Later stages of advanced human prostate cancer are either androgen-insensitive or androgen-repressed. One of the suggested molecular mechanisms that delineates this androgen-repressed phenotype in our EMT model is the expression of phosphorylated androgen-induced proliferation inhibitor (APRIN) in ARCaP cells, as we reported previously [5]. In the present study, we hypothesize another plausible mechanism that involves the interplay of MCM3, MCM7, and androgen receptor (AR). In our EMT ARCaP model, both MCM3 and MCM7 proteins of the replicative DNA helicase were elevated in ARCaP_M_ cells. A recent study indicated that the C-terminus of MCM3 is crucial for recruitment of all MCM2-7 proteins to DNA [12]. This MCM3 tail is required for the loading of the double hexamer on DNA to “license” replication [6]. Cells lacking MCM3 fail to proceed into S phase and proliferate. All of these data suggest MCM3 as an inevitable proliferation marker affecting DNA synthesis and eventually cell division. Several pathways and constituent proteins have been implicated in inducing EMT in cancerous cells. These pathways are typically active in a number of other processes that include cell proliferation, but the link between proliferation-associated proteins like MCM3 and EMT is not well understood [29]. One possibility is that the greater the number of proliferating mesenchymal cells with migratory and invasive phenotype, the more of these aggressive cells are available to metastasize.

One of the most extensively studied MCM proteins, MCM7, is part of DNA helicase and is found to interact with AR [30]. The interaction between AR and MCM7 is vital for gene expression or repressor activity of androgen. This AR/MCM7 complex acts as a co-replication factor affecting cell proliferation and as a co-transcription factor controlling the expression of AR-responsive genes. The availability of androgens inhibits MCM7 expression and causes cell growth arrest. Conversely, the absence of androgens results in an AR/MCM7 interaction that induces the MCM complex to initiate DNA replication [30]. Based on all these findings, we consider that the increased metastatic potential of ARCaP_M_ cells is partially due to increased levels of MCM3 and MCM7, because the more cells proliferate the more cells with EMT phenotype are available for spreading to other parts of the body. Elevated MCM3 and MCM7 proteins are speculated to interact with AR, affecting cell proliferation, migration, and androgen repression. Whether MCM3 interacts directly or indirectly with AR remains to be further investigated.

This study sheds light on EMT and metastasis in human prostate cancer using the ARCaP_E_/ARCaP_M_ cell line model and human prostate cancer tissue specimens. We found an elevated expression of the proliferation marker MCM3 in ARCaP_M_ compared to ARCaP_E_ cells and during the progression of prostate cancer from primary tumor to metastatic disease. This upregulation is hypothesized to involve an interaction with AR either directly or indirectly through MCM7. Collectively, our data suggest a pivotal role of MCM3 in prostate cancer metastasis and a possible AR-MCM7-MCM3 interaction that may be responsible for the growth, metastasis, and androgen-repressed phenotype of late stage human prostate cancer. Understanding the molecular mechanisms and the clinical and pathological parameters of prostate cancer metastasis is of utmost importance to identify new therapeutic targets and improve patient outcomes.

## MATERIALS AND METHODS

### Cell culture and proteomics analysis

Biological triplicates of ARCaP_E_ and ARCaP_M_ were cultured in Dulbecco's Modified Eagle's Medium (Sigma Product # 5523) supplemented with 3.7 grams sodium bicarbonate in a humid incubator at 37°C and 5% CO_2_. Cells were grown until prior to confluence and harvested using a lysis buffer (4% CHAPS, 8M Urea) supplemented with Halt protease and phosphatase inhibitor (ThermoScientific Product # 78443) and scraping. After scraping and collection, lysate was mixed for 45 minutes followed by several freeze-thaw cycles to ensure thorough lysis. Lysate was centrifuged and the supernatant was collected for subsequent proteomic analysis.

Buffer exchange and processing of lysate for proteomic analysis was performed according to a modified filter-aided sample preparation (FASP) protocol with a 30 kDa-cutoff centrifugal filter unit (VWR Product # 82031-352) [31]. Samples were digested with mass spectrometry grade trypsin (ThermoScientific Product # 90059) for 24 hours using a 1:100 enzyme to protein ratio. Protein concentrations of FASP-prepped, digested samples were determined using a bicinchoninic acid assay kit (ThermoScientific Product # 23227), and samples were then uniformly loaded into a nano high performance liquid chromatography column packed with C18 reversed phase silica coupled to an externally calibrated ThermoScientific LTQ Velos nLC-ESI-LIT-Orbitrap (high-resolution electrospray tandem mass spectrometer). nLC-MS/MS was run in technical triplicate for each biological replicate to enable normalization and analysis. The raw files were analyzed using the Proteome Discoverer software package (version 1.4, ThermoScientific, http://www.thermoscientific.com) with SequestHT and Mascot search nodes using species-specific FASTA database and the Percolator peptide validator. The resulting MSF files were further analyzed using the Scaffold proteome validator software (version 4.0.6.1, Proteome Software, Inc., http://www.proteomesoftware.com/).

### Protein expression detection by Western blot

Western blot analysis was performed using cultured cells at 70-80% confluence by previously described protocols [32]. In brief, 20 μg of protein samples were electrophoresed on 4-15% Bis-Tris gradient SDS-PAGE (BioRad 456-1084), transferred onto nitrocellulose membrane (BioRad 162-0115), reacted with 1:200 diluted anti-MCM3 antibody (Cell Signaling # 4012), and followed by secondary goat anti-rabbit antibody conjugated with horseradish peroxidase (Santa Cruz sc-2004). The immune-reactive bands were detected using Kodak Image Station 4000MM Pro instrument (AFAB Lab resources) and Carestream Molecular Imaging Software Network Edition.

### Data analysis

Normalized spectral counts from cell proteomics were assigned to a peptide sequence with 95% confidence. A minimum of two identified peptides was required to confirm a protein sequence with 95% confidence. Missing values were imputed with the minimum non-zero normalized spectral count. An unpaired, two-sample t-test was performed on normalized spectral counts in order to determine differentially expressed proteins between ARCaP_E_ and ARCaP_M_, and a P-value of < 0.05 was used as a cutoff for statistical significance. Pathway analysis was performed using GeneGO MetaCore (Thomson Reuters, https://portal.genego.com/) on proteins with a cut-off mean expression set at least 1.5 fold difference between ARCaP_E_ and ARCaP_M_.

### Immunohistochemistry of prostate cancer patient tissue samples and human tumors grown in nude mice

The immunohistochemistry (IHC) staining method was the same as our previously reported protocol with minor modifications [3, 32]. Formalin-fixed paraffin embedded (FFPE) sections were deparaffinized, rehydrated, and subjected to antigen retrieval. After incubating in Dual Endogenous Enzyme Block solution (Dako, Carpinteria, CA) for 10 minutes, sections were incubated with MCM3 primary antibody (IgG from rabbits) (Cell Signaling # 4012) and diluted with Antibody Diluent solution (Dako) at a 1:50 ratio at room temperature for two hours. The section was then washed 3 times in PBST (phosphate-buffered saline containing 0.2% Tween 20) for 5 minutes per washing and incubated with EnVision+ Dual Link System-HRP (Dako) for 30 minutes, which contains horse radish peroxidase conjugated goat antibodies directed against rabbit IgG. Sections were washed 3 times for 5 minutes each, and stains were developed with 3,3′-diaminobenzidine (Dako). HeLa cells were used as positive control. Negative controls were performed by eliminating the primary antibody or replacing with rabbit IgG prepared at the same concentration and applied to an immediately adjacent tissue section.

## SUPPLEMENTARY TABLES






