# Supplementary material for: Upregulation of minichromosome maintenance complex component 3 during epithelial-to-mesenchymal transition in human prostate cancer
Source: Oncotarget. 2017 Apr 5;8(24):39209–17. doi: 10.18632/oncotarget.16835 (PMC5503607; doi:10.18632/oncotarget.16835)
Supplement: Supplementary file 1 [file oncotarget-08-39209-s001.pdf]

Upregulation of minichromosome maintenance complex component 3 during epithelial-to-mesenchymal transition in human prostate cancer

SUPPLEMENTARY MATERIALS

SUPPLEMENTARY TABLES

Supplementary Table 1: A set of 1,152 proteins were identified in ARCaP epithelial cells and ARCaP mesenchymal cells using proteomic approach

See Supplementary File 1

Supplementary Table 2: Pathway enrichment analysis report for differentially expressed proteins

See Supplementary File 2
